# Supplementary material for: Solution-Processed Smooth Copper Thiocyanate Layer with Improved Hole Injection Ability for the Fabrication of Quantum Dot Light-Emitting Diodes
Source: Nanomaterials (Basel). 2022 Jan 1;12(1):154. doi: 10.3390/nano12010154 (PMC8746833; doi:10.3390/nano12010154)
Supplement: Supplementary file 1 [file nanomaterials-12-00154-s001.zip › nanomaterials-1529144-supplementary.pdf]

Supplementary Materials

Solution-Processed Smooth Copper Thiocyanate Layer with Improved Hole Injection Ability for the Fabrication of Quantum Dot Light-Emitting Diodes

Ming-Ru Wen [1], Sheng-Hsiung Yang [1,*] and Wei-Sheng Chen [2]

[1] Institute of Lighting and Energy Photonics, College of Photonics, National Yang Ming Chiao Tung University, Tainan 71150, Taiwan; lg0895623@gmail.com
[2] Opulence Optronics Co., Ltd., Hsinchu 30091, Taiwan; nabilies.00g@g2.nctu.edu.tw
* Correspondence: yangsh@mail.nctu.edu.tw

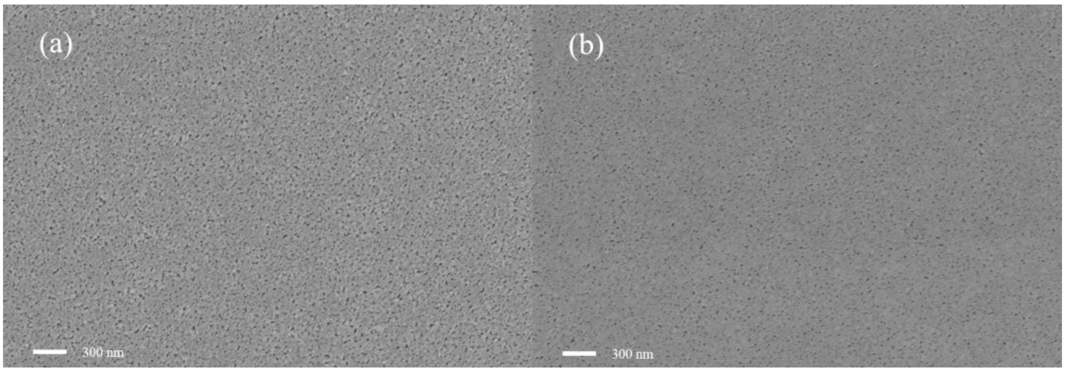

Figure S1. Top-view SEM micrographs of the (a) pristine and (b) F4TCNQ-doped CuSCN films.

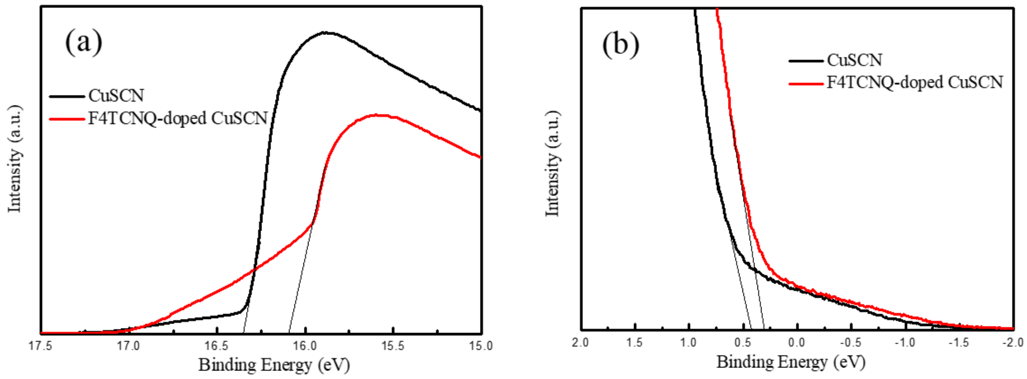

Figure S2. UPS spectra of the pristine and F4TCNQ-doped CuSCN layers in the (a) high-binding energy region and (b) valence-band edge.

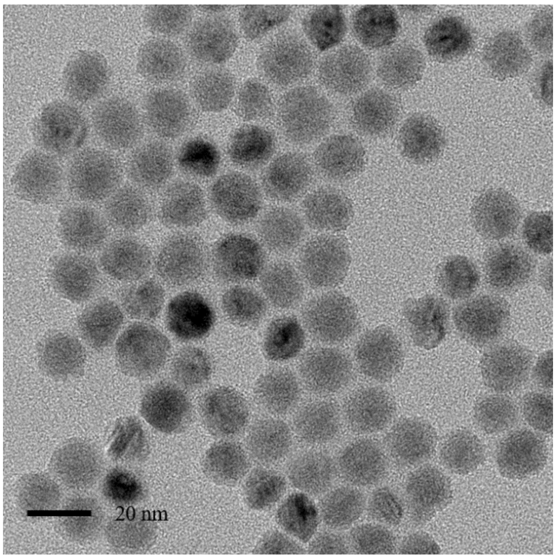

Figure S3. TEM image of CdSe QDs.

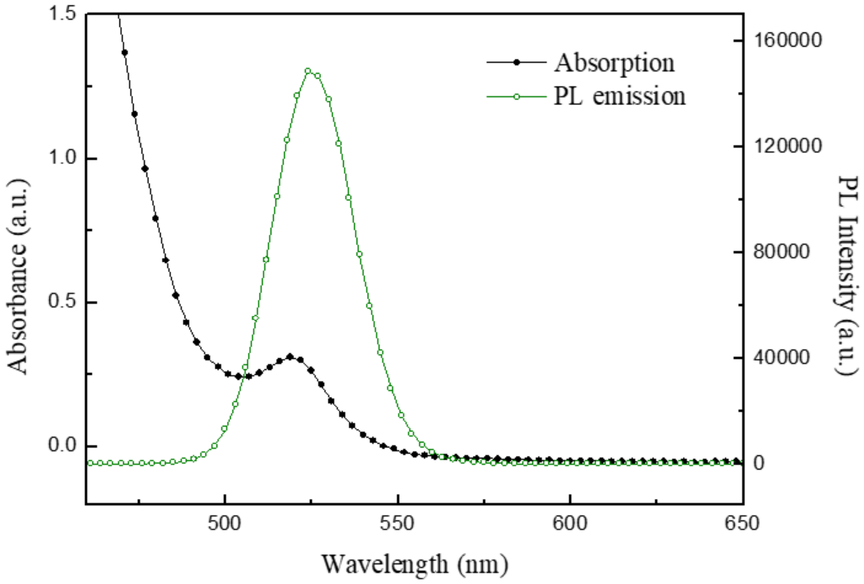

Figure S4. UV-vis absorption and PL emission spectra of CdSe QDs.
